# Evaluation of the prognostic impact of intraoperative cytology results according to tumor sidedness in stage II/III resectable colorectal cancer

**DOI:** 10.1007/s00384-026-05173-z

**Published:** 2026-06-15

**Authors:** Akihiro Usui, Hirotoshi Kobayashi, Kenjiro Kotake, Kotaro Maeda, Takeshi Shuto, Masayasu Kawasaki, Koji Komori, Heita Ozawa, Chihiro Kosugi, Masayuki Ohue, Kimihiko Funahashi, Ichiro Takemasa, Hideyuki Ishida, Shinsuke Kazama, Yoshifumi Shimada, Hajime Motohashi, Yusuke Kinugasa, Yukihide Kanemitsu, Hiroki Ochiai, Soichiro Ishihara, Michio Itabashi, Kenichi Sugihara, Hideki Ueno

**Affiliations:** 1 Department of Surgery, Teikyo University Chiba Medical Center, 3426-3 Anesaki, Ichihara, Chiba, 299-0111 Japan; 2 Department of Surgery, Tokyo Metropolitan Hiroo Hospital, Tokyo, Japan; 3 Department of Surgery, Teikyo University Hospital, Mizonokuchi, Kanagawa Japan; 4 Department of Surgery, Sano City Hospital, Tochigi, Japan; 5 International Medical Center, Fujita Health University Hospital, Toyoake, Japan; 6 Department of Gastroenterological Surgery, Yamagata Prefectural Central Hospital, Yamagata, Japan; 7 Department of Surgery, Bell Land General Hospital, Sakai, Japan; 8 Department of Gastroenterological Surgery, Aichi Cancer Center Hospital, Aichi, Japan; 9 Department of Surgery, Tochigi Cancer Center, Utsunomiya, Japan; 10 Department of Gastroenterological Surgery, Osaka International Cancer Institute, Osaka, Japan; 11 Department of General and Gastroenterological Surgery, Toho University Omori Medical Center, Ota City, Japan; 12 Department of Surgery, Surgical Oncology and Science, Sapporo Medical University, Sapporo, Japan; 13 Department of Digestive Tract and General Surgery, Saitama Medical Center, Saitama Medical University, Kawagoe, Japan; 14 Department of Gastroenterological Surgery, Saitama Cancer Center, Ina-machi, Japan; 15 Division of Digestive and General Surgery, Niigata University Graduate School of Medical and Dental Sciences, Niigata, Japan; 16 Department of Gastroenterological Surgery, Hirosaki University Graduate School of Medicine, Aomori, Japan; 17 Department of Gastrointestinal Surgery, Institute of Science Tokyo, Tokyo, Japan; 18 Department of Colorectal Surgery, National Cancer Center Hospital, Tokyo, Japan; 19 Department of Surgery, Teikyo University School of Medicine, Tokyo, Japan; 20 Department of Surgical Oncology, The University of Tokyo Hospital, Tokyo, Japan; 21 Department of Surgery, Institute of Gastroenterology, Tokyo Women’s Medical University, Tokyo, Japan; 22 Tokyo Medical and Dental University, Tokyo, Japan; 23 Department of Surgery, National Defense Medical College, Tokorozawa, Japan

**Keywords:** Colorectal cancer, Overall survival, Relapse-free survival

## Abstract

**Aim:**

To investigate whether colorectal cancer (CRC) sidedness is associated with intraoperative lavage cytology results, tumor recurrence, and prognosis.

**Method:**

Using data from a multicenter prospective observational study conducted by the Japanese Society for Cancer of the Colon and Rectum (JSCCR), we retrospectively analyzed prognosis and recurrence patterns in pathological stage II/III right-sided and left-sided CRC, stratified by positive versus negative lavage cytology results.

**Results:**

A total of 1500 patients met the inclusion criteria and were enrolled. Of these, 534 had right-sided CRC and 966 had left-sided CRC. Fifty-nine patients (3.9%) had positive lavage cytology. Among patients with recurrence, pT4, positive lavage cytology, and right-sided tumor location were independently associated with peritoneal recurrence.

Among cytology-negative patients, the 5-year relapse-free survival (RFS) rate was significantly higher in right-sided than in left-sided CRC (79.6% vs. 73.4%, *p* = 0.01), whereas the 5-year overall survival (OS) rate did not differ significantly (89.2% vs. 87.7%, *p* = 0.52). Among cytology-positive patients, no statistically significant differences in RFS or OS were observed between tumor locations. However, among cytology-positive patients who developed recurrence, post-recurrence survival was significantly worse in right-sided CRC than in left-sided CRC (*p* = 0.04).

**Conclusion:**

Among cytology-negative patients, left-sided CRC was associated with poorer RFS than right-sided CRC, although OS did not differ. Right-sided tumor location and positive lavage cytology were independently associated with peritoneal recurrence among patients who developed recurrence. Among cytology-positive patients, right-sided CRC was associated with poorer post-recurrence survival.

**Supplementary Information:**

The online version contains supplementary material available at 10.1007/s00384-026-05173-z.

## Introduction

Colorectal cancer (CRC) is the second leading cause of cancer-related mortality in Japan [1] and in Western countries [2]. In Japan, the number of patients with CRC has steadily increased [3]. Although most patients undergo curative resection, recurrence still occurs at a substantial rate. According to data from the Japanese Society for Cancer of the Colon and Rectum (JSCCR), the sites of first recurrence after curative surgery for CRC include the liver (5.7%) and lungs (5.0%), whereas peritoneal dissemination occurs less frequently (2.6%), but is associated with particularly poor outcomes [4]. Advances in systemic chemotherapy for advanced or recurrent unresectable CRC have extended the median survival time (MST) from approximately 8 months to about 30 months [5–8]. However, among the various recurrence sites, peritoneal recurrence is associated with a significantly poorer prognosis than recurrence at other sites [9, 10]. The MST after the diagnosis of peritoneal recurrence is reported to be substantially shorter than that observed for distant recurrence in other organs [10]. Given the poor prognosis of peritoneal recurrence, identification of prognostic factors is critical for guiding appropriate treatment strategies.

The left and right colon originate from distinct embryological regions, suggesting that right- and left-sided CRCs differ in their clinicopathological and biological characteristics [11, 12]. These anatomical and biological differences have been reported to influence prognosis [13–15]. However, no studies to date have examined differences between right- and left-sided CRC in relation to intraoperative peritoneal lavage cytology. In our previous study, we performed intraoperative lavage cytology in patients with CRC and found that positive cytology was associated with a higher incidence of peritoneal recurrence [16]. Building upon these findings, the present study aimed to clarify differences in recurrence patterns and prognosis between cytology-positive and cytology-negative CRC cases in order to support more informed clinical decision-making.

### Study design

This study was a retrospective secondary analysis of a multicenter prospective observational cohort conducted across 20 institutions affiliated with the Japanese Society for Cancer of the Colon and Rectum (JSCCR). Patients who underwent surgery for CRC between October 2012 and December 2016 were enrolled.

The eligibility criteria were as follows: histopathologically confirmed CRC; age ≥ 20 years; clinical stage (cStage) II or III CRC; no history of malignancy within the past 5 years; and provision of written informed consent before surgery. Patients who did not provide preoperative informed consent were excluded. Patients with pathological stage 0–I disease were also excluded because the present study specifically focused on recurrence patterns and long-term prognosis in stage II/III CRC, in which postoperative recurrence is clinically relevant. Eligibility and data completeness were centrally verified by the JSCCR Data Center using predefined case registration criteria and periodic data monitoring procedures. Surgical procedures were not standardized by the study protocol and were performed at the discretion of each participating institution. Prognostic data were collected for at least 3 years after surgery. Patient characteristics included age, sex, and tumor stage. Written informed consent was obtained from all participants prior to enrollment. The study was conducted in accordance with the principles of the Declaration of Helsinki and ethical guidelines for clinical research. This study was registered with the UMIN Clinical Trials Registry (UMIN000026070).

### Data collection

All data were collected prospectively. Preoperative data included demographic characteristics, blood test results, and clinical staging information such as tumor depth and lymph node status. Information on postoperative adjuvant chemotherapy and survival outcomes was collected for at least 3 years after surgery. Patients underwent blood tests and computed tomography (CT) every 3 months postoperatively to monitor for recurrence. Follow-up data were collected prospectively at participating institutions for at least 3 years postoperatively. Precise reasons for loss to follow-up were not available in the database. The median follow-up duration was 64.0 months. Adjuvant chemotherapy was administered according to institutional protocols and attending physicians’ discretion. Detailed information regarding chemotherapy regimens, treatment duration, and completion rates was not uniformly available in the database. Post-recurrence survival was defined as the time from diagnosis of recurrence to death from any cause, with patients alive at last follow-up censored.

### Tumor sidedness definition

Right-sided CRC was defined as tumors located from the cecum to the splenic flexure, whereas left-sided CRC included tumors distal to the splenic flexure, including rectal cancer. This classification was based on embryological and anatomical laterality used in previous CRC sidedness studies [11, 12].

### Statistical analysis

Differences in continuous variables were compared using Student’s *t*-test, and differences in categorical variables were analyzed using the chi-square test. OS was defined as the time from surgery to death from any cause, with patients alive at last follow-up censored. RFS was defined as the time from surgery to first recurrence or death from any cause, with patients alive without recurrence at last follow-up censored. Survival curves were estimated using the Kaplan–Meier method and compared using the log-rank test. Multivariate Cox proportional hazards analyses were performed to identify independent prognostic factors for RFS and OS. Variables included age, sex, histological type, pathological T stage, pathological stage, cytology status, and tumor sidedness. Multivariate logistic regression analysis was performed to identify factors associated with peritoneal recurrence among patients who developed recurrence. Variables included age, sex, histological type, pathological T stage, pathological stage, lymphatic invasion, venous invasion, preoperative treatment, cytology status, and tumor sidedness. Because adjuvant chemotherapy was a postoperative treatment variable determined by pathological risk factors and institutional discretion, and because detailed information regarding regimen, duration, and completion was unavailable, it was not included in the primary model. Therefore, adjuvant chemotherapy was not included in the multivariate model and was addressed as a limitation. Propensity score matching was not performed because the relatively small number of cytology-positive cases would have markedly reduced statistical power and matching reliability. A *p* value < 0.05 was considered statistically significant, and 95% confidence intervals (CIs) were calculated. All statistical analyses were performed using SPSS software (version 22.0; SPSS Inc., Chicago, IL, USA).

## Results

### Patient characteristics

**Fig. 1:**
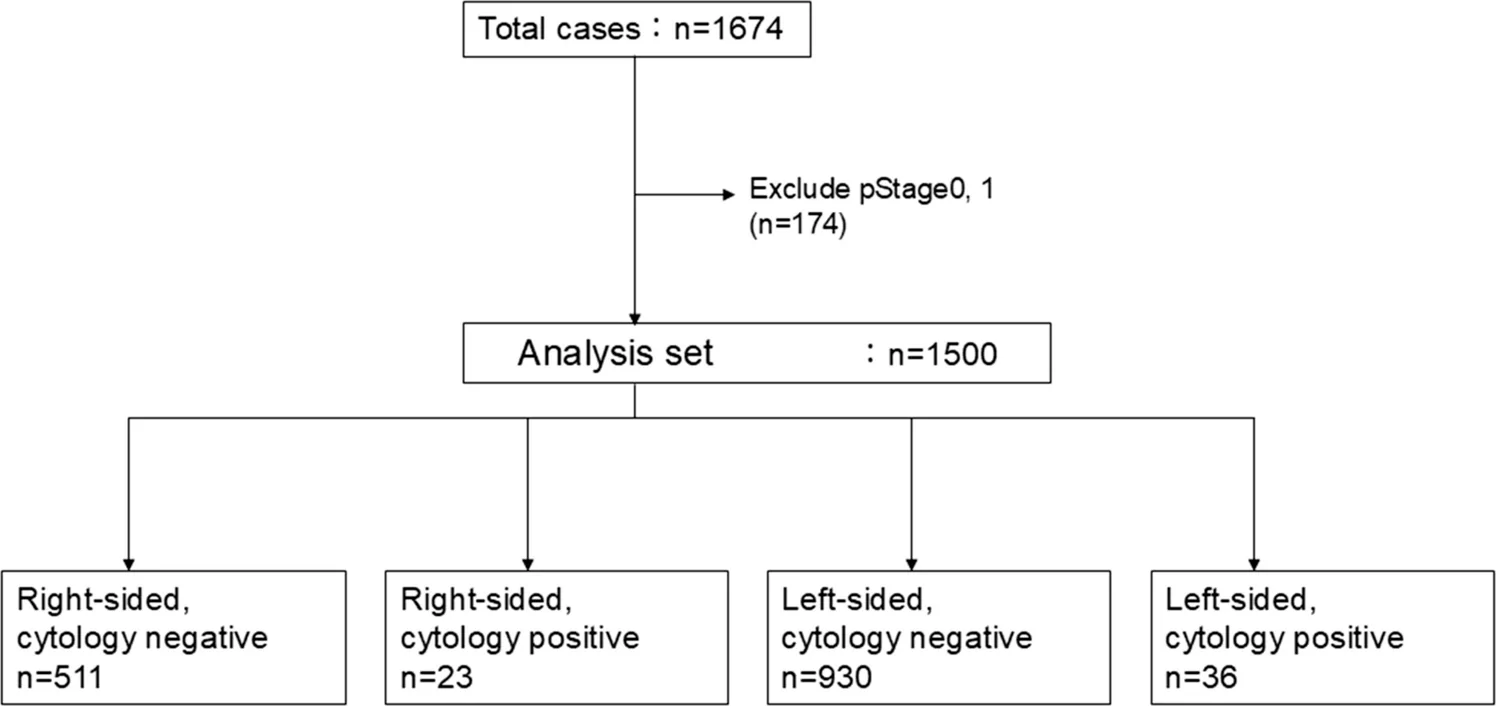
Patient selection

The data flow for this study is presented in Fig. 1. A total of 1674 patients were enrolled, of whom 174 were excluded because they were diagnosed with pathological stage 0–I disease after surgery. Ultimately, 1500 patients with pathological stage II/III CRC were included in the analysis.

### Lavage cytology

Among the 1500 patients, 59 (3.9%) had positive lavage cytology—23 with right-sided CRC and 36 with left-sided CRC—while 1441 (96.1%) had negative cytology—511 with right-sided CRC and 930 with left-sided CRC (Fig. 1).

The characteristics of patients with right- and left-sided CRC, stratified by cytology status, are summarized in Tables 1 and 2. Variables included age, sex, tumor location, histological type, pathological stage, preoperative treatment, and postoperative adjuvant chemotherapy.

**Table 1:**
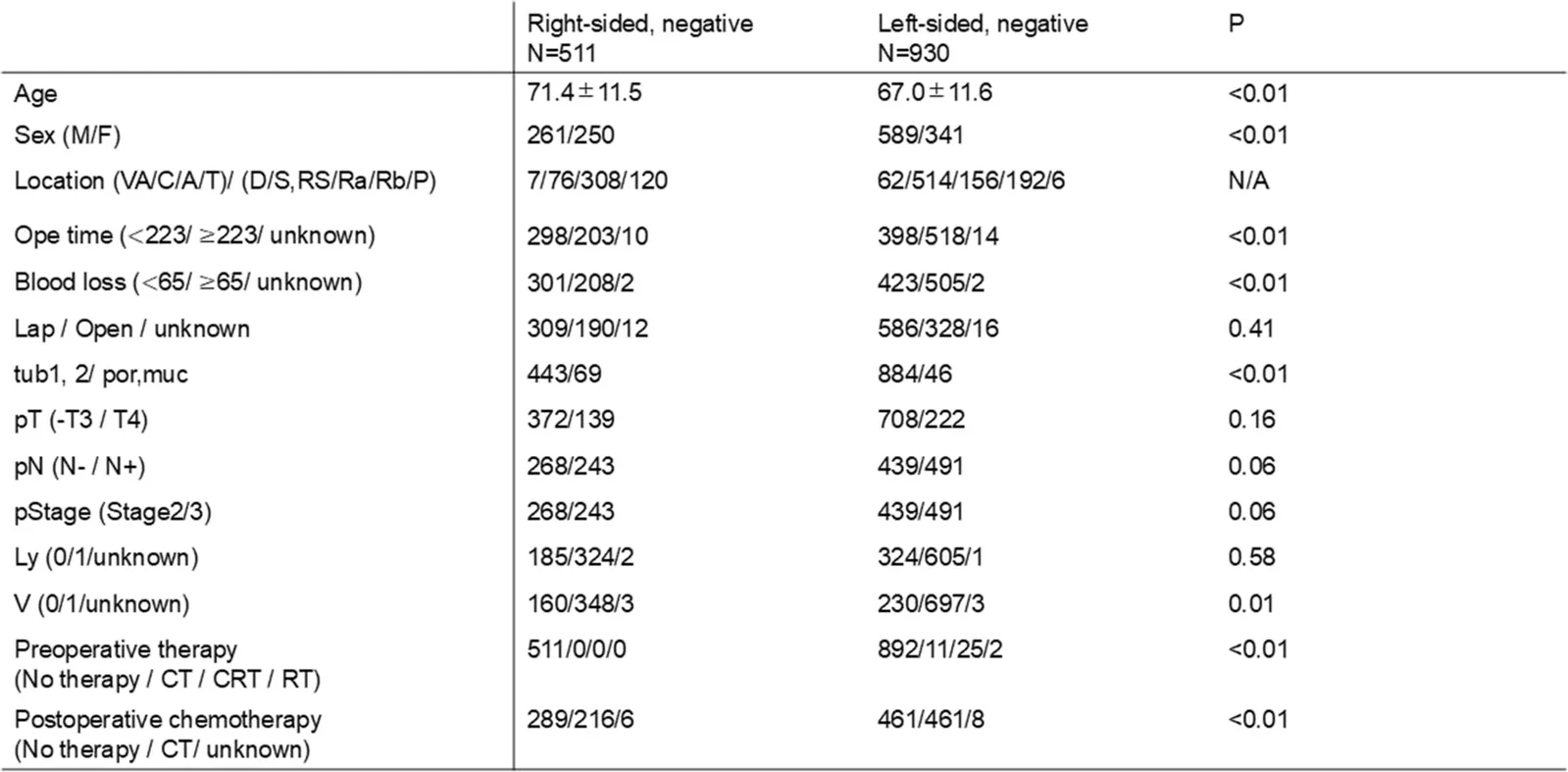
Characteristics of patients with right- and left-sided CRC in cytology-negative cases

*CRC* colorectal cancer, *VA* vermiform appendix, *C* cecum, *A* ascending colon, *T* transverse colon, *D* descending colon, *S* sigmoid colon, *RS* rectosigmoid, *Ra* upper rectum above the peritoneal reflection, *Rb* lower rectum below the peritoneal reflection, *P* anal canal, *tub1* well-differentiated adenocarcinoma, *tub2* moderately differentiated adenocarcinoma, *por* poorly differentiated adenocarcinoma, *muc* mucinous adenocarcinoma, *Ly* lymphatic invasion, *V* venous invasion, *CT* chemotherapy, *CRT* chemoradiotherapy, *RT* radiotherapy

**Table 2:**
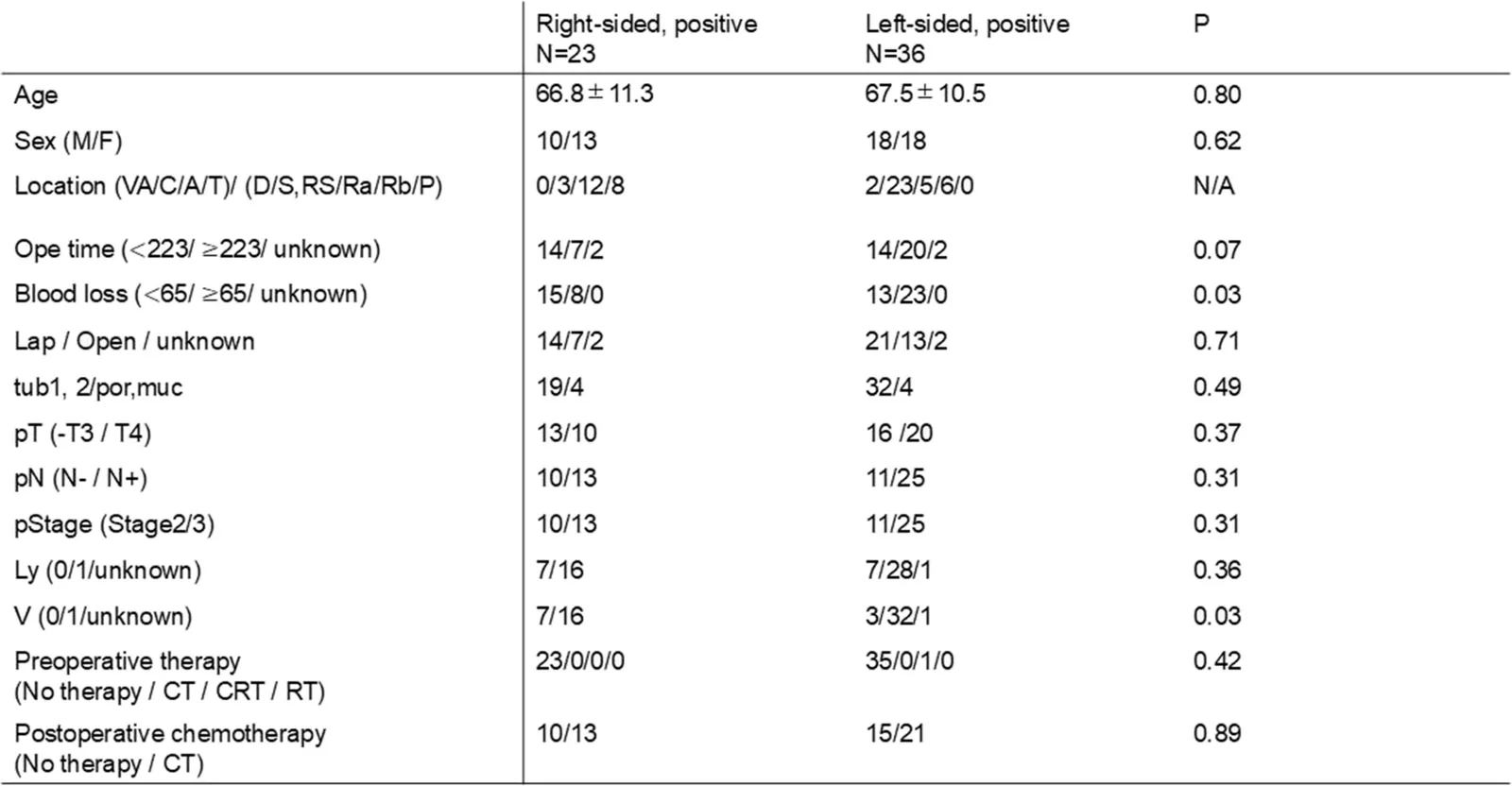
Characteristics of patients with right- and left-sided CRC in cytology-positive cases

Abbreviations are as defined in Table 1.

### Survival

**Fig. 2:**
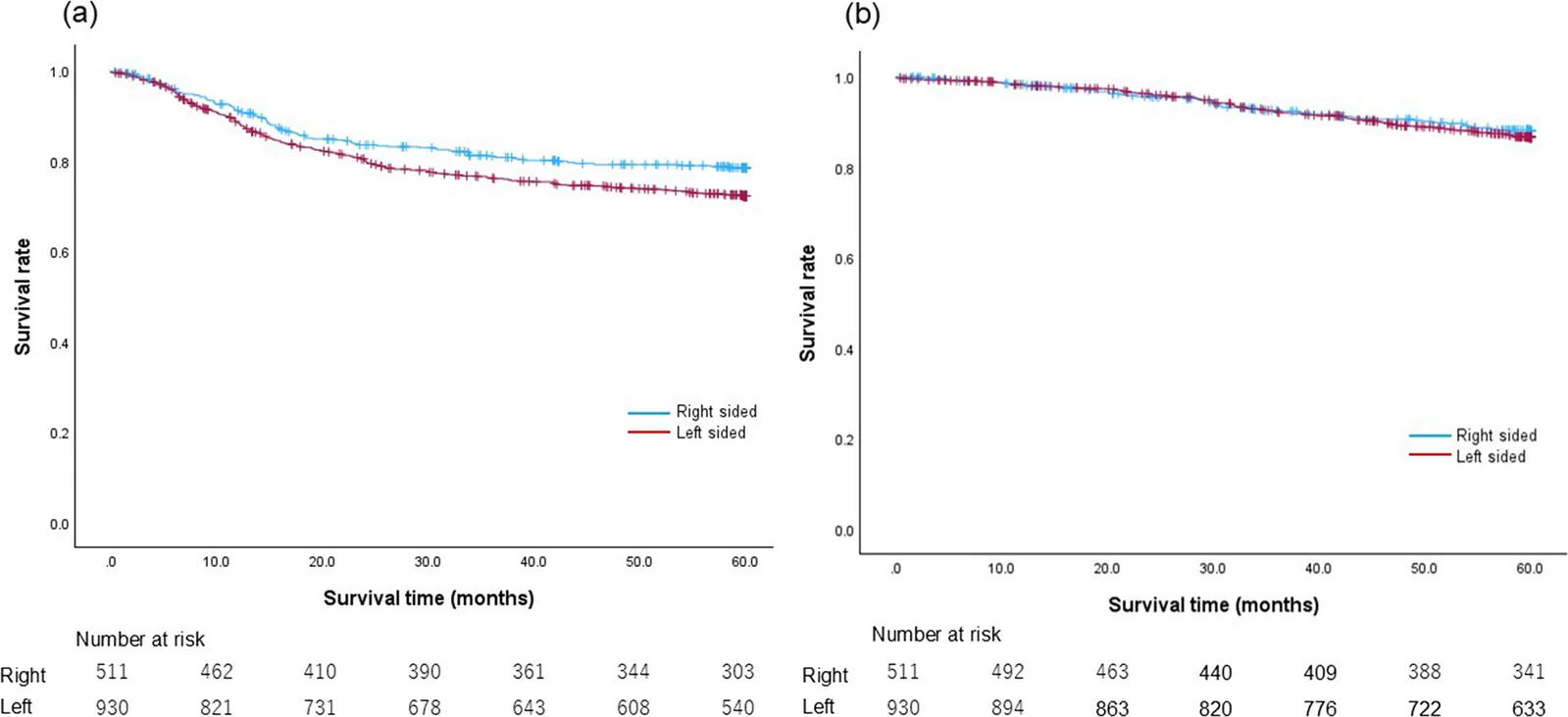
Kaplan–Meier curves for cytology-negative cases. **a** Relapse-free survival. **b** Overall survival

In cytology-negative cases, the 5-year relapse-free survival (RFS) rates for right-sided and left-sided CRC were 79.6% and 73.4%, respectively (*p* = 0.01), while the corresponding 5-year overall survival (OS) rates were 89.2% and 87.7% (*p* = 0.52) (Fig. 2).

**Fig. 3:**
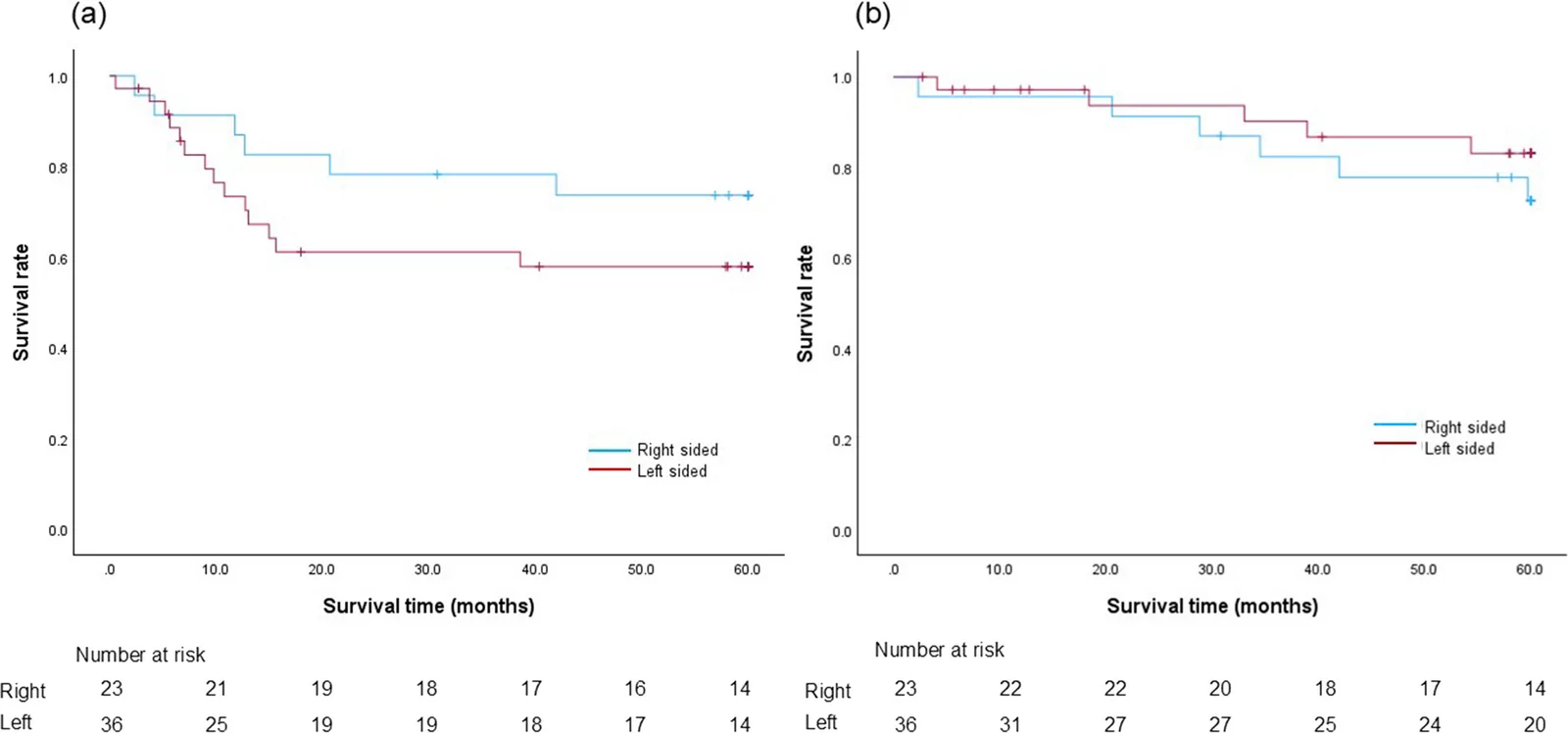
Kaplan–Meier curves for cytology-positive cases. **a** Relapse-free survival. **b** Overall survival

In cytology-positive cases, the 5-year RFS rates for right-sided and left-sided CRC were 73.9% and 61.1%, respectively (*p* = 0.21), and the 5-year OS rates were 73.9% and 86.1%, respectively (*p* = 0.40) (Fig. 3).

Among cytology-negative patients, right-sided CRC showed significantly better RFS than left-sided CRC (*p* = 0.01), whereas OS did not differ significantly (*p* = 0.52). In cytology-positive patients, neither RFS nor OS differed significantly between right- and left-sided CRC.

In a multivariate Cox proportional hazards model including age, sex, histological type, pathological T stage, pathological stage, cytology status, and tumor sidedness, left-sidedness remained independently associated with poorer RFS (HR 1.367, 95% CI 1.086–1.721, *p* = 0.008) (Supplementary Table 1a). In the corresponding model for OS, tumor sidedness was not independently associated with OS (HR 1.157, 95% CI 0.842–1.591, *p* = 0.369) (Supplementary Table 1b).

### Location of recurrence

Among cytology-negative patients, recurrence rates for right-sided and left-sided CRC were 17.2% and 23.7%, respectively. Among cytology-positive patients, recurrence rates were 26.1% and 38.9%, respectively (Table 3).

**Table 3:**
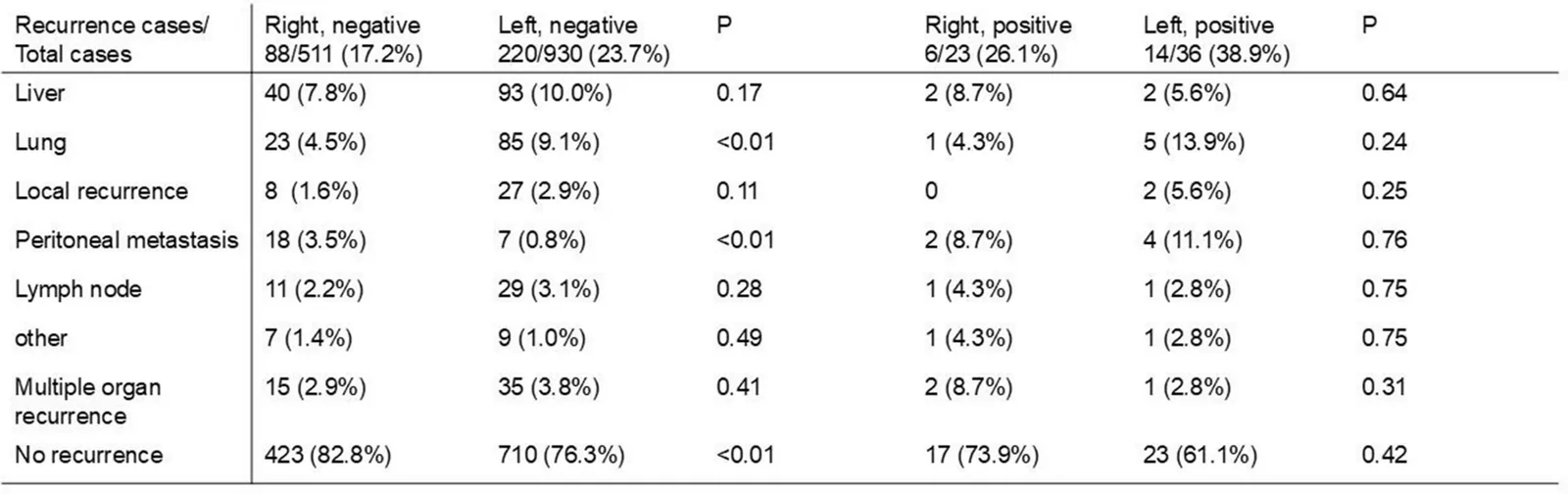
Sites of recurrence according to tumor sidedness and lavage cytology status

In cytology-negative cases, right-sided CRC was associated with significantly more peritoneal metastases than left-sided CRC (*p* < 0.01), whereas left-sided CRC was associated with significantly more lung metastases (*p* < 0.01) (Table 3).

### Peritoneal recurrence

We analyzed factors associated with peritoneal recurrence among patients who developed recurrence. In multivariate analysis, pT4, positive IPLC, and right-sided tumor location were independently associated with peritoneal recurrence (*p* = 0.012, *p* = 0.036, and *p* = 0.014, respectively) (Table 4).

**Table 4:**
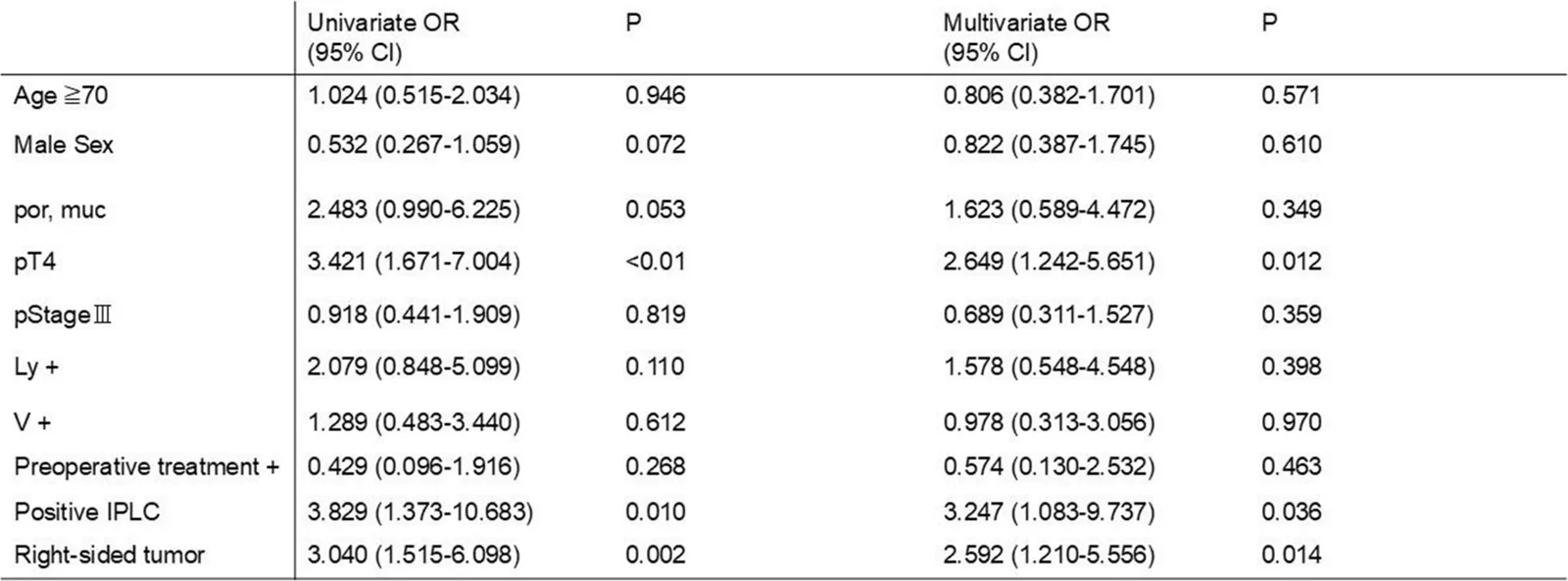
Factors associated with peritoneal recurrence among patients with recurrence

*OR* odds ratio, *CI* confidence interval, *IPLC* intraoperative peritoneal lavage cytology, *Ly* lymphatic invasion, *V* venous invasion

**Fig. 4:**
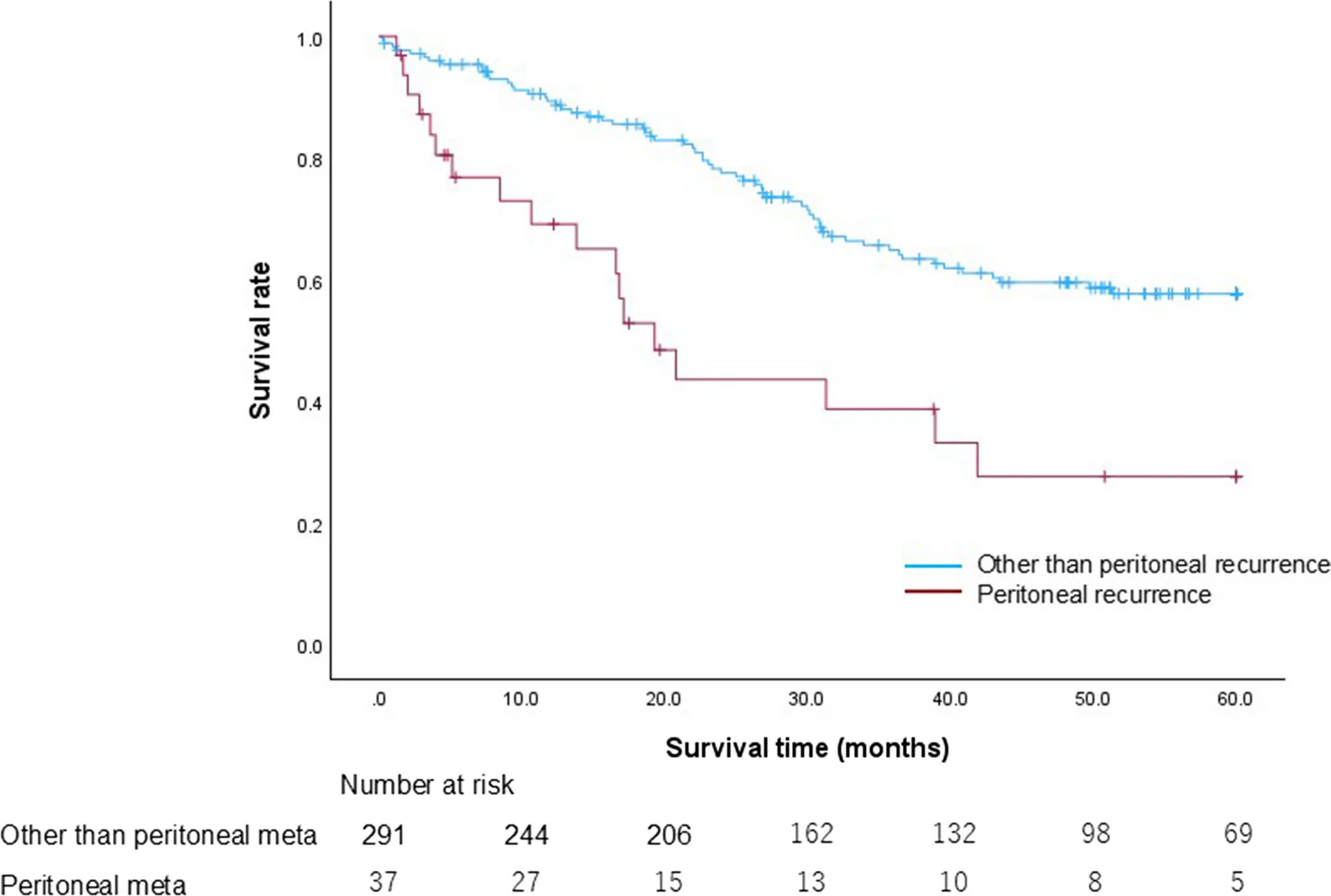
Post-recurrence survival according to recurrence site. Post-recurrence survival was compared between patients with peritoneal recurrence and those with recurrence at other sites

To assess the clinical implications of peritoneal recurrence, survival after recurrence was compared between patients with peritoneal recurrence and those with recurrence at other sites. The 5-year post-recurrence survival rate was 43.8% in patients with peritoneal recurrence and 64.0% in those with recurrence at other sites (Fig. 4). Patients with peritoneal recurrence had a significantly poorer prognosis than those with recurrence at other sites (*p* < 0.001).

### Treatment after recurrence

Table 5 presents the initial treatments administered after recurrence. The highest proportion of patients receiving potentially curative surgical treatment was observed in the group with left-sided CRC and negative cytology (54.1%), followed by those with right-sided CRC and negative cytology (43.2%), left-sided CRC and positive cytology (35.7%), and right-sided CRC and positive cytology (16.7%).

**Table 5:**
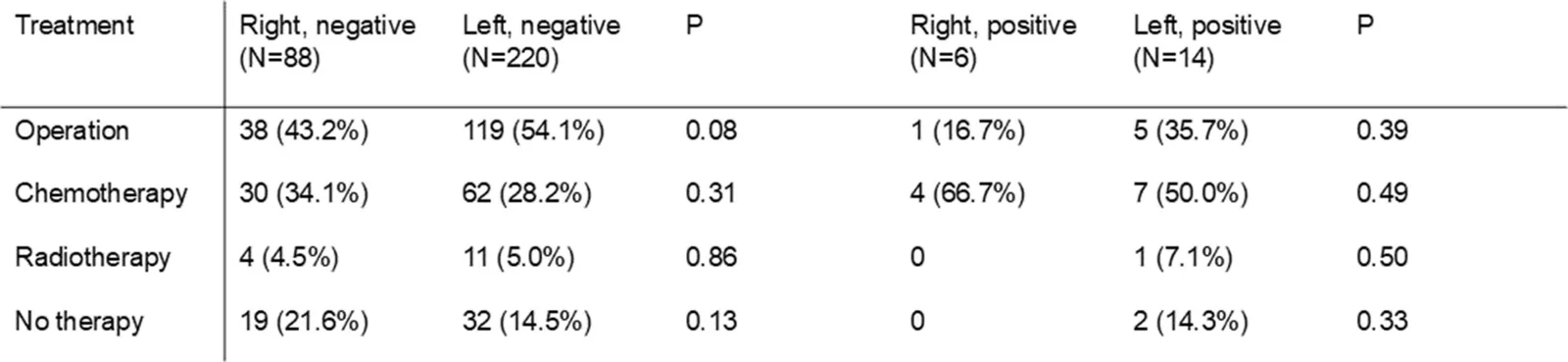
Initial treatment after recurrence

**Fig. 5:**
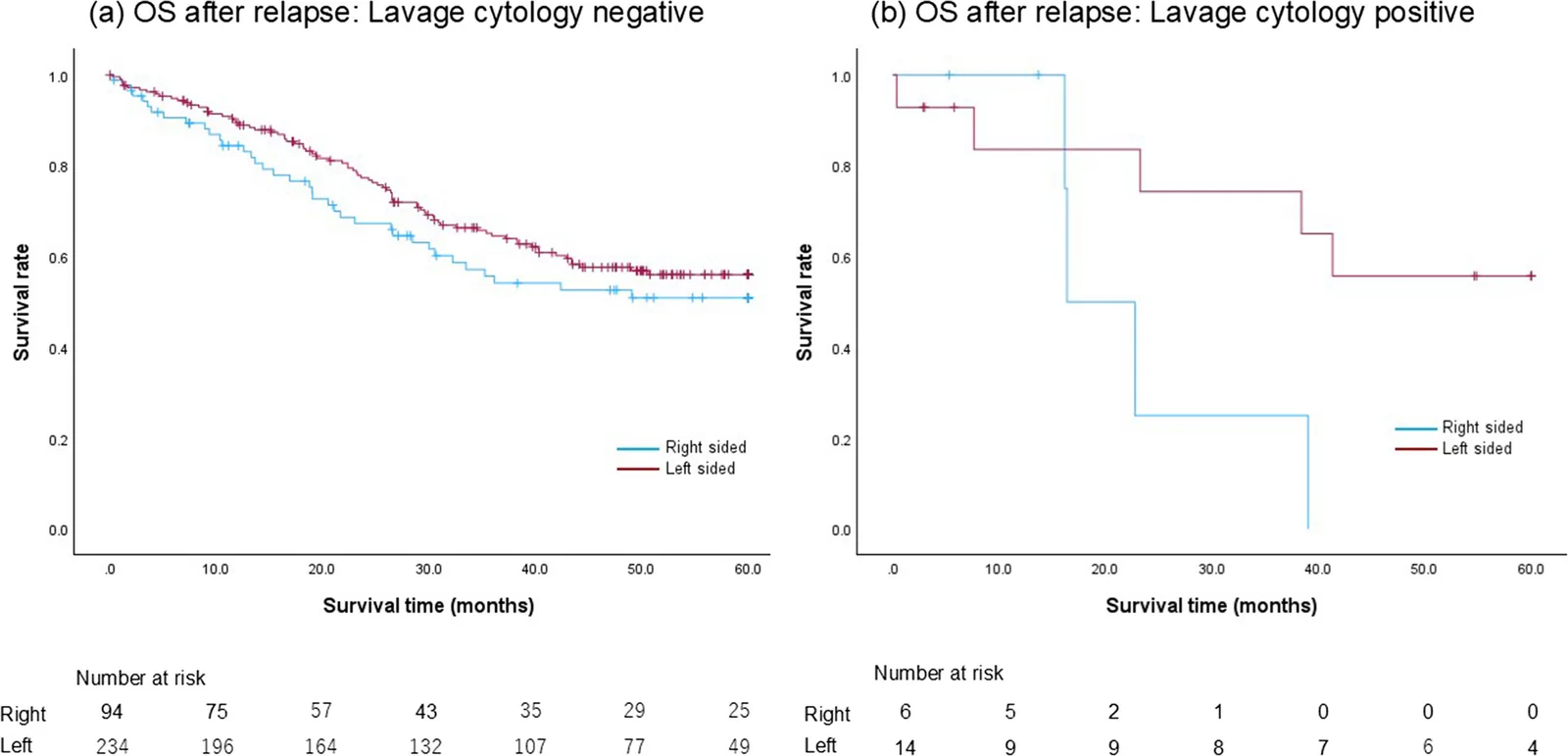
Post-recurrence survival according to tumor sidedness and lavage cytology status. **a** Post-recurrence survival in cytology-negative cases. **b** Post-recurrence survival in cytology-positive cases

Among patients with negative cytology, the 5-year post-recurrence survival rates were 57.5% for right-sided CRC and 62.1% for left-sided CRC (*p* = 0.25). Among patients with positive cytology, the 5-year post-recurrence survival rates were 33.3% for right-sided CRC and 64.3% for left-sided CRC (*p* = 0.04) (Fig. 5).

## Discussion

In the present study, among cytology-negative patients, those with left-sided CRC had worse 5-year RFS than those with right-sided CRC, although no difference was observed in 5-year OS. These findings suggest that patients with left-sided CRC were more likely to experience recurrence but were less likely to die after recurrence compared with patients with right-sided CRC. A plausible explanation is the higher proportion of peritoneal recurrence among patients with right-sided CRC, which is linked to a poor post-recurrence prognosis. In our analysis, right-sided tumor location remained independently associated with peritoneal recurrence, together with pT4 and positive IPLC. Because peritoneal recurrence is associated with significantly worse survival than recurrence at other sites, patients with right-sided CRC, which was independently associated with peritoneal recurrence in the present analysis, may have poorer outcomes after recurrence. Furthermore, consistent with previous reports, the cytology-positive group demonstrated a high incidence of peritoneal recurrence [16].

The prognostic significance of tumor sidedness has been investigated in several studies. Right- and left-sided CRCs differ in embryological origin, clinicopathological features, molecular background, and clinical outcomes [11–15]. Right-sided CRC has been reported to be more common in older patients and women and to show distinct metastatic patterns compared with left-sided CRC [12, 15]. In particular, differences in recurrence sites, including peritoneal, liver, and lung metastases, may partly explain the distinct post-recurrence outcomes observed in the present study [15].

In the cytology-positive group, as in the cytology-negative group, 5-year RFS was lower in left-sided CRC than in right-sided CRC, although the difference was not statistically significant (Fig. 3). Because the cytology-positive cohort included patients who did and did not receive adjuvant chemotherapy, and detailed regimen information was unavailable, the influence of adjuvant treatment on survival could not be fully assessed. This trend may be influenced by the small number of cytology-positive cases, which limits statistical power. These patterns are reflected in post-recurrence survival curves. Among cytology-negative patients, post-recurrence survival tended to be better for left-sided CRC than right-sided CRC, although the difference was not significant. In cytology-positive patients, post-recurrence survival was significantly better for left-sided CRC than for right-sided CRC. Thus, cytology-positive patients with right-sided CRC may have an unfavorable post-recurrence prognosis.

According to the JSCCR guidelines, adjuvant chemotherapy is recommended for patients with stage III CRC and may be considered for selected high-risk stage II patients [4]. Several clinical trials have supported the use of fluoropyrimidine- and oxaliplatin-based adjuvant chemotherapy in stage III colon cancer [17–19]. High-risk features for stage II CRC include T4 tumors, inadequate lymph node evaluation, poor differentiation, lymphovascular invasion, perineural invasion, obstruction or perforation, and positive surgical margins [4, 20]. However, detailed information regarding adjuvant chemotherapy regimens, duration, and treatment completion was not uniformly available in the present dataset.

Given these results, cytology-positive patients with right-sided CRC may represent a particularly high-risk subgroup. Therefore, careful postoperative risk assessment is important in this subgroup. These findings suggest that patients with right-sided CRC and positive cytology may require closer postoperative surveillance, particularly for peritoneal recurrence.

This study has some limitations. First, because this was a multi-institutional observational study, treatment strategies, including the indications and duration of adjuvant chemotherapy, varied among institutions. Detailed information regarding adjuvant chemotherapy regimens, duration, and treatment completion was not uniformly available, which may have influenced recurrence and survival outcomes. However, because all participating institutions are members of the JSCCR, a certain level of treatment quality is likely to have been maintained. Future studies should standardize the indications and duration of adjuvant chemotherapy. Adjuvant chemotherapy was not included in the primary multivariate model for peritoneal recurrence for this reason and because its administration was not randomized and was strongly influenced by pathological stage and recurrence risk. Therefore, including adjuvant chemotherapy as a simple covariate may have introduced confounding by indication. Secondly, cases of right- and left-sided CRC differed in baseline characteristics such as age and sex, suggesting the need for propensity score matching. However, this approach was not adopted because the number of cases with positive cytology was extremely small, and matching would have further reduced the sample size. Based on these findings, a prospective clinical study with a larger number of cytology-positive cases is warranted. Third, rectal cancer was included in the left-sided CRC group. Although this classification is commonly used in studies of CRC sidedness, rectal cancer may differ from colon cancer in terms of treatment strategy, local recurrence risk, and metastatic pattern. Because the number of cytology-positive patients was limited, separate subgroup analysis of left-sided colon cancer and rectal cancer was not feasible. Fourth, the number of cytology-positive patients was small, which limited the statistical power of subgroup analyses and increased the possibility of type II error. Fifth, molecular information, including MSI status and RAS/BRAF mutation status, was not available. These factors may influence recurrence patterns and prognosis according to tumor sidedness.

## Conclusion

In conclusion, tumor sidedness was associated with distinct recurrence patterns and prognostic characteristics according to intraoperative lavage cytology status in patients with stage II/III CRC. Right-sided tumor location and positive lavage cytology were independently associated with peritoneal recurrence among patients who developed recurrence. In addition, patients with right-sided CRC and positive cytology had poorer post-recurrence survival. These findings suggest that this subgroup may represent a high-risk population that warrants careful postoperative surveillance, particularly for peritoneal recurrence.

## Supplementary Information

Below is the link to the electronic supplementary material.

ESM 1 (PDF 559 KB)

ESM 2 (PDF 274 KB)

ESM 3 (JPG 141 KB)

## Data Availability

The datasets analyzed during the current study are available from the corresponding author upon reasonable request.

## References

[1] Matsuda T, Fujimoto A, Igarashi Y (2025) Colorectal cancer: epidemiology, risk factors, and public health strategies. Digestion 106:91–99. 10.1159/000543921 39938491 10.1159/000543921

[2] Siegel RL, Giaquinto AN, Jemal A (2024) Cancer statistics, 2024. CA Cancer J Clin 74:12–49. 10.3322/caac.21820 38230766 10.3322/caac.21820

[3] Muto T, Kotake K, Koyama Y (2001) Colorectal cancer statistics in Japan: data from JSCCR registration, 1974–1993. Int J Clin Oncol 6:171–176. 10.1007/PL00012102 11706554 10.1007/pl00012102

[4] Hashiguchi Y, Muro K, Saito Yea et al (2020) Japanese Society for Cancer of the Colon and Rectum (JSCCR) guidelines 2019 for the treatment of colorectal cancer. Int J Clin Oncol 25:1–42. 10.1007/s10147-019-01485-z 31203527 10.1007/s10147-019-01485-z PMC6946738

[5] Yamada Y, Takahari D, Matsumoto Hea et al (2013) Leucovorin, fluorouracil, and oxaliplatin plus bevacizumab versus S-1 and oxaliplatin plus bevacizumab in patients with metastatic colorectal cancer (SOFT): an open-label, non-inferiority, randomised phase 3 trial. Lancet Oncol 2045(13):1278–1286. 10.1016/S1470-2045(13)70490-X 10.1016/S1470-2045(13)70490-X 24225157

[6] Yamazaki K, Nagase M, Tamagawa Hea et al (2016) Randomized phase III study of bevacizumab plus FOLFIRI and bevacizumab plus mFOLFOX6 as first-line treatment for patients with metastatic colorectal cancer (WJOG4407G). Ann Oncol 27:1539–1546. 10.1093/annonc/mdw206 27177863 10.1093/annonc/mdw206

[7] Loupakis F, Cremolini C, Masi Gea et al (2014) Initial therapy with FOLFOXIRI and bevacizumab for metastatic colorectal cancer. N Engl J Med 371:1609–1618. 10.1056/nejmoa1403108 25337750 10.1056/NEJMoa1403108

[8] Watanabe J, Muro K, Shitara K et al (2023) Panitumumab vs bevacizumab added to standard first-line chemotherapy and overall survival among patients with RAS wild-type, left-sided metastatic colorectal cancer: a randomized clinical trial. JAMA 329:1271–1282. 10.1001/jama.2023.4428 37071094 10.1001/jama.2023.4428 PMC10114040

[9] Quere P, Facy O, Manfredi Sea et al (2015) Epidemiology, management, and survival of peritoneal carcinomatosis from colorectal cancer. Dis Colon Rectum 58:743–752. 10.1097/DCR.0000000000000412 26163953 10.1097/DCR.0000000000000412

[10] Van Gestel YRBM, Thomassen I, Lemmens Vea et al (2014) Metachronous peritoneal carcinomatosis after curative treatment of colorectal cancer. Eur J Surg Oncol 40:963–969. 10.1016/j.ejso.2013.10.001 24183168 10.1016/j.ejso.2013.10.001

[11] Iacopetta B (2002) Are there two sides to colorectal cancer? Int J Cancer 101:403–408 12216066 10.1002/ijc.10635

[12] Mik M, Berut M, Dziki L et al (2017) Right-and left-sided colon cancer-clinical and pathological differences of the disease entity in one organ. Arch Med Sci 13:157–162. 10.5114/aoms.2016.58596 28144267 10.5114/aoms.2016.58596 PMC5206358

[13] Yaqi L, Yang F, Weixing D, Qingguo L, Sanjun C, Peng J (2019) Prognostic effect of tumor sidedness in colorectal cancer: a SEER-based analysis. Clin Colorectal Cancer 18(1):e104–e116. 10.1016/j.clcc.2018.10.005 30448100 10.1016/j.clcc.2018.10.005

[14] Brulé SY, Jonker DJ, Karapetis CS et al (2015) Location of colon cancer (right-sided versus left-sided) as a prognostic factor and a predictor of benefit from cetuximab in NCIC CO.17. Eur J Cancer 51:1405–1414. 10.1016/j.ejca.2015.03.015 25979833 10.1016/j.ejca.2015.03.015

[15] Waldstein S, Spengler M, Pinchuk IV, Yee NS (2023) Impact of colorectal cancer sidedness and location on therapy and clinical outcomes: role of blood-based biopsy for personalized treatment. J Pers Med. 10.3390/jpm13071114 37511727 10.3390/jpm13071114 PMC10381730

[16] Kobayashi H, Kotake K, Maeda K et al (2024) Peritoneal lavage cytology in patients with curative resection for stage II and III colorectal cancer: a multi-institutional prospective study. Ann Gastroenterol Surg 8:807–816. 10.1002/ags3.12825 39229555 10.1002/ags3.12825 PMC11368490

[17] André T, Meyerhardt J, Iveson T et al (2020) Effect of duration of adjuvant chemotherapy for patients with stage III colon cancer (IDEA collaboration): final results from a prospective, pooled analysis of six randomised, phase 3 trials. Lancet Oncol 21:1620–1629. 10.1016/S1470-2045(20)30527-1 33271092 10.1016/S1470-2045(20)30527-1 PMC7786835

[18] Haller DG, Tabernero J, Maroun Jea et al (2011) Capecitabine plus oxaliplatin compared with fluorouracil and folinic acid as adjuvant therapy for stage III colon cancer. J Clin Oncol 29:1465–1471. 10.1200/JCO.2010.33.6297 21383294 10.1200/JCO.2010.33.6297

[19] Kuebler JP, Wieand HS, O’Connell MJ et al (2007) Oxaliplatin combined with weekly bolus fluorouracil and leucovorin as surgical adjuvant chemotherapy for stage II and III colon cancer: results from NSABP C-07. J Clin Oncol 25:2198–2204. 10.1200/JCO.2006.08.2974 17470851 10.1200/JCO.2006.08.2974

[20] Sadahiro S, Sakamoto K, Tsuchiya T, Sakamoto J et al (2022) Prospective observational study of the efficacy of oral uracil and tegafur plus leucovorin for stage II colon cancer with risk factors for recurrence using propensity score matching (JFMC46–1201). BMC Cancer 22(1):170. 10.1186/s12885-022-09267-z 35168560 10.1186/s12885-022-09267-z PMC8845390

