# Supplementary material for: Evaluation of the prognostic impact of intraoperative cytology results according to tumor sidedness in stage II/III resectable colorectal cancer
Source: Int J Colorectal Dis. 2026 Jun 15;41(1):140. doi: 10.1007/s00384-026-05173-z (PMC13500429; doi:10.1007/s00384-026-05173-z)
Supplement: Supplementary file 1 [file 384_2026_5173_MOESM1_ESM.pdf]

IJCD􏰀 2.docx

Teikyo University - Itabashi Campus

文書の詳細

提出ID

trn:oid:::3117:580238126

提出日

2026年4月19日 8:45 JST

ダウンロード日

2026年4月19日 9:53 JST

ファイル名

IJCD􏰀 2.docx

ファイルサイズ

38.1 KB

12 ページ

3,382 語

21,615 文字

11% 全体的な類似性

各データベースに対する、重複するソースを含む全ての一致の合計

レポートからフィルタ済み

- ビブリオグラフィー
- 引用符のあるテキスト
- 引用されたテキスト
- 小さな一致（12ワード未満）
- Methods and Materials

除外

- 5除外された一致

一致グループ

17 引用および引用符なし 11%
テキスト内の引用も引用符もない一致

0 引用符なし 0%
ソース文書に非常に類似する一致

0 引用なし 0%
引用符はあるものの、テキスト内の引用がない一致

0 引用および引用符あり 0%
テキスト内の引用はあるものの、引用符がない一致

上位のソース

10% インターネットソース
9% 出版物
0% 提出物（学生レポート）

インテグリティフラグ

レビューの対象となる0個のインテグリティフラグ

不審なテキスト操作は見つかりませんでした。

システムのアルゴリズムによって、ドキュメントに通常の答案と異なる不一致がないか詳細に調査されます。不審な点が見つかれば、レビューできるようにフラグが付けられます。

フラッグは必ずしも問題を示すものではありませんが、詳細を確認することをお勧めします。

一致グループ

- **17 引用および引用符なし 11%**
  テキスト内の引用も引用符もない一致
- **0 引用符なし 0%**
  ソース文書に非常に類似する一致
- **0 引用なし 0%**
  引用符はあるものの、テキスト内の引用がない一致
- **0 引用および引用符あり 0%**
  テキスト内の引用はあるものの、引用符がない一致

上位のソース

10% インターネットソース
9% 出版物
0% 提出物（学生レポート）

上位のソース

答案内で一致件数が最も多いソース。重複するソースは表示されません。

1	インターネット	www.ncbi.nlm.nih.gov	1%
2	インターネット	link.springer.com	1%
3	インターネット	research.tees.ac.uk	1%
4	インターネット	www.aaccjnls.org	<1%
5	出版物	C.H. Maeng, A. Alkashash, A. Sy, H. Barnes, S. Bannon, C. Simms, M.R. Strickland, J....	<1%
6	インターネット	pubmed.ncbi.nlm.nih.gov	<1%
7	インターネット	thejns.org	<1%
8	インターネット	www.researchsquare.com	<1%
9	インターネット	pastel.archives-ouvertes.fr	<1%
10	出版物	Seo Ae Han, Jae Hyun Kim, Ji Hun Choi, Do Hyeong Lee, Kyoungwon Jung, Sung Eu...	<1%

11 インターネット

www.frontiersin.org <1%

12 出版物

Nobukazu Agatsuma, Takahiro Utsumi, Yoshitaka Nishikawa, Takahiro Horimats... <1%

13 出版物

Takahiro Hiratsuka, Tsuyoshi Etoh, Takao Hara, Tomonori Akagi et al. "Long-term... <1%

14 インターネット

www.pubfacts.com <1%

15 出版物

Tomoki Nakamura, Akira Kawai, Kunihiro Asanuma, Tomohito Hagi, Akihiro Sudo.... <1%

Title: Evaluation of the Prognostic Impact of Intraoperative Cytology Results According to Tumor Sidedness in Stage II/III Resectable Colorectal Cancer

Authors:
Akihiro Usui, MD[1], Hirotoshi Kobayashi, MD[2,3], Kenjiro Kotake, MD[4], Kotaro Maeda, MD[5], Takeshi Shuto, MD[6], Masayasu Kawasaki, MD[7], Koji Komori, MD[8], Heita Ozawa MD[9], Chihiro Kosugi, MD[1], Masayuki Ohue, MD[10], Kimihiko Funabashi, MD[11], Ichiro Takemasa, MD[12], Hideyuki Ishida, MD[13], Shinsuke Kazama, MD[14], Yoshifumi Shimada, MD[15], Hajime Motohashi, MD[16], Yusuke Kinugasa, MD[17], Yukihide Kanemitsu, MD[18], Hiroki Ochiai, MD[19], Soichiro Ishihara, MD[20], Michio Itabashi, MD[21], Kenichi Sugihara, MD[22], Hideki Ueno, MD[23]

[1] Department of Surgery, Teikyo University Chiba Medical Center, Ichihara City, Chiba, Japan.
[2] Department of Surgery, Tokyo Metropolitan Hiroo Hospital, Tokyo, Japan.
[3] Department of Surgery, Teikyo University Hospital, Mizonokuchi, Kanagawa, Japan.
[4] Department of Surgery, Sano City Hospital, Tochigi, Japan.
[5] International Medical Center, Fujita Health University Hospital, Toyoake, Japan.
[6] Department of Gastroenterological Surgery, Yamagata Prefectural Central Hospital, Yamagata, Japan.
[7] Department of Surgery, Bell Land General Hospital, Sakai, Japan.
[8] Department of Gastroenterological Surgery, Aichi Cancer Center Hospital, Aichi, Japan.
[9] Department of Surgery, Tochigi Cancer Center, Utsunomiya, Japan.
[10] Department of Gastroenterological Surgery, Osaka International Cancer Institute, Osaka, Japan.
[11] Department of General and Gastroenterological Surgery, Toho University Omori Medical Center, Japan.
[12] Department of Surgery, Surgical Oncology and Science, Sapporo Medical University, Sapporo, Japan.
[13] Department of Digestive Tract and General Surgery, Saitama Medical Center, Saitama Medical University, Kawagoe, Japan.
[14] Department of Gastroenterological Surgery, Saitama Cancer Center, Japan.
[15] Division of Digestive and General Surgery, Niigata University Graduate School of Medical and Dental Sciences, Niigata, Japan.
[16] Department of Gastroenterological Surgery, Hirosaki University Graduate School of Medicine, Aomori, Japan.
[17] Department of Gastrointestinal Surgery, Institute of Science Tokyo, Tokyo, Japan.

[18] Department of Colorectal Surgery, National Cancer Center Hospital, Tokyo, Japan.
[19] Department of Surgery, Teikyo University School of Medicine, Tokyo, Japan.
[20] Department of Surgical Oncology, The University of Tokyo Hospital, Tokyo, Japan.
[21] Department of Surgery, Institute of Gastroenterology, Tokyo Women's Medical University, Tokyo, Japan.
[22] Tokyo Medical and Dental University, Tokyo, Japan.
[23] Department of Surgery, National Defense Medical College, Tokorozawa, Japan.

Address for Correspondence: Akihiro Usui, MD, PhD
Department of Surgery, Teikyo University Chiba Medical Center, 3426-3 Anesaki, Ichihara, Chiba, 299-0111, Japan
Tel:+81-436-62-1211
Fax:+81-436-61-3961
E-mail:
usui.akihiro.gj@teikyo-u.ac.jp
ausui@med.teikyo-u.ac.jp

3

Funding Declaration:
This research received no external funding.

Data Availability:
The datasets analyzed during the current study are available from the corresponding author upon reasonable request.

Conflicts of Interest:
The authors declare no conflict of interest.

Abstract

Aim:

This study aimed to clarify whether tumor sidedness in colorectal cancer (CRC) influences intraoperative lavage cytology findings, recurrence patterns, and long-term outcomes.

Methods:

11 We performed a secondary analysis of a multicenter prospective dataset collected by the Japanese Society for Cancer of the Colon and Rectum (JSCCR). Patients with pathological stage II/III CRC were categorized by tumor sidedness and lavage cytology status, and survival outcomes and recurrence patterns were compared. This study adhered to the STROBE guidelines for reporting (checklist provided).

Results:

Among 1,500 eligible patients, 534 had right-sided and 966 had left-sided CRC. Positive lavage cytology was identified in 59 patients (3.9%). In recurrent cases, right-sided tumors were more frequently associated with peritoneal relapse ($p = 0.04$).

In the cytology-negative cohort, right-sided CRC showed a higher 5-year relapse-free survival (RFS) rate than left-sided CRC (79.6% vs. 73.4%, $p = 0.01$), whereas overall survival (OS) was comparable (89.2% vs. 87.7%, $p = 0.52$).

In the cytology-positive cohort, no statistically significant differences in RFS or OS were observed between tumor locations. However, among patients who developed recurrence, right-sided CRC was associated with significantly worse survival after recurrence ($p = 0.04$).

Conclusion:

Tumor sidedness impacts recurrence behavior and prognosis in CRC, particularly in relation to cytology status. Right-sided CRC with positive cytology was characterized by frequent peritoneal recurrence and poor post-recurrence survival.

Statement:

Colorectal cancer sidedness was associated with distinct recurrence patterns and prognosis according to lavage cytology status. Cytology-negative patients with left-sided CRC showed poorer relapse-free survival than those with right-sided CRC, whereas overall survival was similar. Cytology-positive patients with right-sided CRC more frequently developed peritoneal recurrence and had poorer post-recurrence outcomes than those with left-sided CRC.

Introduction

Colorectal cancer (CRC) remains a major cause of cancer-related death worldwide and is the second leading cause of cancer-related mortality in Japan [1] and in Western countries [2]. The incidence of CRC in Japan continues to increase [3]. Although curative resection can be achieved in many patients, postoperative recurrence still represents a major clinical problem.

Patterns of recurrence after curative surgery vary, with metastases commonly involving the liver (5.7%) and lungs (5.0%), whereas peritoneal dissemination occurs less frequently (2.6%) but is associated with particularly poor outcomes [4]. Advances in systemic chemotherapy have markedly improved survival in patients with unresectable or recurrent CRC, extending the median survival time from approximately 8 months to around 30 months [5–8]. However, peritoneal recurrence remains associated with significantly worse prognosis compared with metastases to other organs [9,10]. The median survival after peritoneal recurrence is reported to be substantially shorter than that observed in other metastatic settings [10]. These findings highlight the need to identify patients at high risk of peritoneal dissemination.

Colorectal tumors differ according to their embryological origin, with the right and left colon arising from distinct developmental backgrounds [11,12]. Right-sided CRC, defined as tumors located between the cecum and the splenic flexure, and left-sided CRC, located distal to the splenic flexure, have been shown to exhibit differences in clinicopathological and molecular characteristics, which may influence prognosis [13–15].

Despite these differences, the association between tumor sidedness and intraoperative peritoneal lavage cytology has not been well characterized. In our previous study, positive lavage cytology was associated with an increased risk of peritoneal recurrence [16]. Based on these findings, the present study aimed to evaluate how tumor sidedness interacts with cytology status to influence recurrence patterns and survival outcomes in patients with stage II/III CRC.

Study design

This was a multicenter, prospective observational study conducted across 20 institutions affiliated with the Japanese Society for Cancer of the Colon and Rectum (JSCCR). Patients who underwent surgical resection for CRC between October 2012 and December 2016 were consecutively enrolled.

Eligible patients met the following criteria: histologically confirmed CRC, age ≥20 years, clinical stage II or III disease, no malignancy within the preceding 5 years, and provision of written informed consent prior to surgery. Patients lacking preoperative consent were excluded.

Eligibility was centrally verified through the JSCCR Data Center. Surgical procedures and perioperative management were determined by each participating institution. Patients were followed for at least 3 years postoperatively. The study was conducted in accordance with the Declaration of Helsinki and approved ethical standards, and was registered in the UMIN Clinical Trials Registry (UMIN000026070).

Data collection

All data were collected prospectively. Preoperative data included demographic characteristics, blood test results, and clinical staging information such as tumor depth and lymph node status. Information on postoperative adjuvant chemotherapy and survival outcomes was collected for at least 3 years after surgery. Patients underwent blood tests and computed tomography (CT) every 3 months postoperatively to monitor for recurrence.

Statistical analysis

Continuous variables were evaluated using the Student's t-test, while categorical variables were compared with the chi-square test.

Overall survival (OS) was defined as the interval from surgery to death from any cause, and relapse-free survival (RFS) as the time from surgery to recurrence or death. Survival distributions were estimated using the Kaplan-Meier method and compared with the log-rank test.

All statistical tests were two-sided, and a p-value < 0.05 was considered statistically significant. Analyses were performed using SPSS version 22.0.

Results

Patient characteristics

The data flow for this study is presented in Fig. 1. A total of 1,674 patients were enrolled, of whom 174 were excluded because they were diagnosed with pathological Stage 0–I disease after surgery. Ultimately, 1,500 patients with pathological Stage II/III CRC were

included in the analysis.

Lavage cytology

Among the 1,500 patients, 59 (3.9%) had positive lavage cytology (23 with right-sided CRC and 36 with left-sided CRC), whereas 1,441 (96.1%) had negative cytology (511 and 930 patients, respectively) (Fig. 1).

The characteristics of patients with right- and left-sided CRC, stratified by cytology status, are summarized in Table 1a–b. Variables included age, sex, tumor location, histological type, pathological stage, preoperative treatment, and postoperative adjuvant chemotherapy.

Survival

In cytology-negative cases, the 5-year relapse-free survival (RFS) rates for right-sided and left-sided CRC were 79.6% and 73.4%, respectively ($p = 0.01$), while the corresponding 5-year overall survival (OS) rates were 89.2% and 87.7% ($p = 0.52$) (Fig. 2).

In cytology-positive cases, the 5-year RFS rates for right-sided and left-sided CRC were 73.9% and 61.1%, respectively ($p = 0.21$), and the 5-year OS rates were 86.1% and 73.9%, respectively ($p = 0.40$) (Fig. 3).

Among cytology-negative patients, right-sided CRC showed significantly better RFS than left-sided CRC ($p = 0.01$), whereas OS did not differ significantly ($p = 0.52$). In cytology-positive patients, neither RFS nor OS differed significantly between right- and left-sided CRC.

Location of recurrence

Among cytology-negative patients, recurrence rates for right-sided and left-sided CRC were 17.2% and 23.7%, respectively. Among cytology-positive patients, recurrence rates were 26.1% and 38.9%, respectively (Table 2).

In cytology-negative cases, right-sided CRC was associated with significantly more peritoneal metastases than left-sided CRC ($p < 0.01$), whereas left-sided CRC was associated with significantly more lung metastases ($p < 0.01$) (Table 2).

Peritoneal recurrence

We analyzed factors associated with peritoneal recurrence. Univariate analysis identified right-sided CRC and pathological T stage as significant predictors; however, in multivariate analysis, only right-sided CRC remained significant ($p = 0.04$) (Table 3).

To assess the clinical implications of peritoneal recurrence, survival after recurrence was

compared between patients with peritoneal recurrence and those with recurrence at other sites. The 5-year post-recurrence survival rate was 43.8% in patients with peritoneal recurrence and 64.0% in those with recurrence at other sites (Fig. 4). Patients with peritoneal recurrence had a significantly poorer prognosis than those with recurrence at other sites ($p < 0.01$).

Treatment after recurrence

Table 5 presents the initial treatments administered after recurrence. The highest proportion of patients receiving potentially curative surgical treatment was observed in the group with left-sided CRC and negative cytology (54.1%), followed by those with right-sided CRC and negative cytology (43.0%), left-sided CRC and positive cytology (35.7%), and right-sided CRC and positive cytology (16.7%) (Table 4).

Among patients with negative cytology, the 5-year post-recurrence survival rates were 57.5% for right-sided CRC and 62.1% for left-sided CRC ($p = 0.25$). Among patients with positive cytology, the 5-year post-recurrence survival rates were 33.3% for right-sided CRC and 64.3% for left-sided CRC ($p = 0.04$) (Fig. 5).

Discussion

In the present study, we demonstrated that tumor sidedness influences recurrence patterns and prognosis in colorectal cancer (CRC), particularly when stratified by intraoperative lavage cytology status. Among cytology-negative patients, left-sided CRC was associated with significantly poorer relapse-free survival (RFS) compared with right-sided CRC, whereas overall survival (OS) did not differ. This discrepancy suggests that although recurrence may occur more frequently in left-sided tumors, survival after recurrence is relatively preserved.

One possible explanation for this finding is the difference in metastatic patterns according to tumor location. In our analysis, right-sided CRC was independently associated with peritoneal recurrence, which is known to confer a particularly poor prognosis [9,10]. In contrast, left-sided CRC is more likely to metastasize to the liver and lungs [15], where multimodal treatment strategies, including surgical resection, are more often feasible. These differences may contribute to the observed survival patterns, in which recurrence is more frequent in left-sided CRC but outcomes after recurrence are less unfavorable than in right-sided CRC.

The prognostic significance of tumor sidedness has been widely reported. Park et al. demonstrated that the 5-year local recurrence rate was higher in right-sided CRC than in left-sided CRC [16]. Conversely, several studies have suggested that early-stage right-

sided CRC may be associated with better outcomes, potentially due to a higher prevalence of microsatellite instability-high (MSI-H) tumors [17]. Furthermore, a meta-analysis of 65 studies reported that left-sided CRC was associated with a significantly lower risk of death compared with right-sided CRC [18]. These findings indicate that the prognostic impact of tumor sidedness is complex and may vary depending on disease stage and clinical context. In the cytology-positive subgroup, no statistically significant differences in RFS or OS were observed between right- and left-sided CRC, likely due to the limited number of patients. However, among patients who developed recurrence, right-sided CRC was associated with significantly poorer post-recurrence survival. This observation suggests that the combination of right-sided tumor location and positive cytology identifies a subgroup of patients with particularly aggressive disease biology.

Consistent with our previous report, cytology-positive patients demonstrated a high incidence of peritoneal recurrence [16]. Given that peritoneal dissemination is associated with markedly worse survival compared with metastases to other organs [9,10], the presence of free cancer cells detected by intraoperative lavage cytology may reflect occult peritoneal disease. This is particularly relevant in right-sided CRC, which was identified as an independent risk factor for peritoneal recurrence in the present study.

Differences in treatment after recurrence may also have influenced survival outcomes. In this study, a higher proportion of patients with left-sided CRC underwent surgical treatment for recurrent disease, and all patients who received such treatment survived following the intervention. This likely contributed to the relatively favorable post-recurrence survival observed in left-sided CRC. In contrast, patients with right-sided CRC, particularly those with positive cytology, were less likely to undergo curative-intent treatment after recurrence, resulting in poorer outcomes.

Currently, the JSCCR guidelines recommend adjuvant chemotherapy for patients with stage III CRC and for selected high-risk stage II cases [17–19]. High-risk features include factors such as T4 tumors, poor differentiation, vascular or lymphatic invasion, and insufficient lymph node evaluation [20]. Based on the findings of the present study, cytology positivity—especially in right-sided CRC—may represent an additional high-risk factor for recurrence and poor prognosis.

These results suggest that intraoperative lavage cytology may provide clinically meaningful prognostic information in patients with advanced CRC. In particular, patients with right-sided CRC and positive cytology appear to be at extremely high risk of peritoneal recurrence and poor survival after recurrence. For such patients, more intensive postoperative surveillance and consideration of adjuvant treatment strategies may be warranted.

This study has several limitations. First, as a multicenter observational study, treatment strategies—including indications for and duration of adjuvant chemotherapy—were not standardized across institutions, which may have introduced variability in outcomes. However, because all participating institutions are affiliated with the JSCCR, a certain level of consistency in treatment quality can be assumed. Second, baseline characteristics differed between right- and left-sided CRC, including age and sex. Although propensity score matching could have addressed this imbalance, it was not performed due to the small number of cytology-positive cases, which would have further reduced statistical power. Further large-scale prospective studies are needed to validate these findings, particularly in patients with positive cytology. Clarification of the clinical significance of intraoperative cytology may contribute to improved risk stratification and optimization of treatment strategies in colorectal cancer.

Conclusion

Among cytology-negative patients, left-sided CRC was associated with a poorer RFS than right-sided CRC. In contrast, cytology-positive patients with right-sided CRC more frequently developed peritoneal recurrence and had a poorer post-recurrence prognosis than those with left-sided CRC. In advanced colorectal cancer, intraoperative cytology may be beneficial, and adjuvant chemotherapy should be considered in cytology-positive patients with right-sided CRC to improve survival outcomes.

4

Acknowledgements:

This study was performed as a part of a project study of the JSCCR.

Legends:

Figure 1: Patient selection

Table 1a: The characteristics of patients with right- and left-sided CRC (cytology-negative cases)

V, vermiform appendix; C, cecum; A, ascending colon;

T, transverse colon; RSJ, rectosigmoid junction;

13 upper rectum (Ra), rectum above the peritoneal reflection;

lower rectum (Rb), rectum below the peritoneal reflection;

anal canal (P)

8 Tub1; well differentiated adenocarcinoma, tub2; moderately differentiated adenocarcinoma, por; poorly differentiated adenocarcinoma, muc; mucinous adenocarcinoma

8 Table 1b: The characteristics of patients with right- and left-sided CRC (cytology-positive cases)

6 Figure 2: Kaplan-Meier curves in cytology-negative cases
a: Relapse-free survival. b: Overall survival

Figure 3: Kaplan-Meier curves in cytology-positive cases
a: Relapse-free survival b: Overall survival

Table 2: Location of recurrence

Table 3: Risk factors for peritoneal recurrence for curative resection in recurrent cases

Figure 4: Comparison of prognosis between peritoneal recurrence and recurrence in other sites after recurrence

Table 4: First treatment for recurrent cases

Figure 5: Post-recurrence survival rates
a: Overall survival after relapse in lavage cytology negative
b: Overall survival after relapse in lavage cytology positive

Reference:

1. Matsuda T, Fujimoto A, Igarashi Y (2025) Colorectal Cancer: Epidemiology, Risk Factors, and Public Health Strategies. Digestion 106:91–99. https://doi.org/10.1159/000543921
2. Siegel RL, Giaquinto AN, Jemal A (2024) Cancer statistics, 2024. CA Cancer J Clin 74:12–49. https://doi.org/10.3322/caac.21820
3. Muto T, Kotake K, Koyama Y, et al (1974) CANCER STATISTICS
4. Hashiguchi Y, Muro K, Saito Y, et al (2020) Japanese Society for Cancer of the Colon and Rectum (JSCCR) guidelines 2019 for the treatment of colorectal cancer. Int J Clin Oncol 25:1–42. https://doi.org/10.1007/s10147-019-01485-z
5. Yamada Y, Takahari D, Matsumoto H, et al (2013) Leucovorin, fluorouracil, and oxaliplatin plus bevacizumab versus S-1 and oxaliplatin plus bevacizumab in patients with metastatic colorectal cancer (SOFT): An open-label, non-inferiority, randomised phase 3 trial. Lancet Oncol 14:1278–1286. https://doi.org/10.1016/S1470-2045(13)70490-X

6. Yamazaki K, Nagase M, Tamagawa H, et al (2016) Randomized phase III study of bevacizumab plus FOLFIRI and bevacizumab plus mFOLFOX6 as first-line treatment for patients with metastatic colorectal cancer (WJOG4407G). Annals of Oncology 27:1539–1546. https://doi.org/10.1093/annonc/mdw206
7. Loupakis F, Cremolini C, Masi G, et al (2014) Initial Therapy with FOLFOXIRI and Bevacizumab for Metastatic Colorectal Cancer. New England Journal of Medicine 371:1609–1618. https://doi.org/10.1056/nejmoa1403108
8. Watanabe J, Muro K, Shitara K, et al (2023) Panitumumab vs Bevacizumab Added to Standard First-line Chemotherapy and Overall Survival among Patients with RAS Wild-type, Left-Sided Metastatic Colorectal Cancer: A Randomized Clinical Trial. JAMA 329:1271–1282. https://doi.org/10.1001/jama.2023.4428
9. Quere P, Facy O, Manfredi S, et al (2015) Epidemiology, management, and survival of peritoneal carcinomatosis from colorectal cancer: A population-based study. Dis Colon Rectum 58:743–752. https://doi.org/10.1097/DCR.0000000000000412
10. Van Gestel YRBM, Thomassen I, Lemmens VEPP, et al (2014) Metachronous peritoneal carcinomatosis after curative treatment of colorectal cancer. European Journal of Surgical Oncology 40:963–969. https://doi.org/10.1016/j.ejso.2013.10.001
11. Iacopetta B (2002) Are there two sides to colorectal cancer? Int. J. Cancer 101:403–408
12. Mik M, Berut M, Dziki L, et al (2017) Right-and left-sided colon cancer-clinical and pathological differences of the disease entity in one organ. Archives of Medical Science 13:157–162. https://doi.org/10.5114/aoms.2016.58596
13. Yaqi Li 1, Yang Feng 2, Weixing Dai 1, Qingguo Li 1, Sanjun Cai 1 JP 3 (2019) Prognostic Effect of Tumor Sidedness in Colorectal Cancer: A SEER-Based Analysis. Clin Colorectal Cancer 18(1):e104-e116. https://doi.org/10.1016/j.clcc.2018.10.005
14. Brulé SY, Jonker DJ, Karapetis CS, et al (2015) Location of colon cancer (right-sided versus left-sided) as a prognostic factor and a predictor of benefit from cetuximab in NCIC CO.17. Eur J Cancer 51:1405–1414. https://doi.org/10.1016/j.ejca.2015.03.015
15. Waldstein S, Spengler M, Pinchuk I V., Yee NS (2023) Impact of Colorectal Cancer Sidedness and Location on Therapy and Clinical Outcomes: Role of Blood-Based Biopsy for Personalized Treatment. J. Pers. Med. 13
16. Kobayashi H, Kotake K, Maeda K, et al (2024) Peritoneal lavage cytology in patients with curative resection for stage II and III colorectal cancer: A multi-institutional prospective study. Ann Gastroenterol Surg 8:807–816. https://doi.org/10.1002/ags3.12825
17. André T, Meyerhardt J, Iveson T, et al (2020) Effect of duration of adjuvant chemotherapy for patients with stage III colon cancer (IDEA collaboration): final results from a prospective, pooled analysis of six randomised, phase 3 trials. Lancet Oncol 21:1620–1629.

https://doi.org/10.1016/S1470-2045(20)30527-1
18. Haller DG, Tabernero J, Maroun J, et al (2011) Capecitabine plus oxaliplatin compared with fluorouracil and folinic acid as adjuvant therapy for stage III colon cancer. Journal of Clinical Oncology 29:1465–1471. https://doi.org/10.1200/JCO.2010.33.6297
19. Kuebler JP, Wieand HS, O'Connell MJ, et al (2007) Oxaliplatin combined with weekly bolus fluorouracil and leucovorin as surgical adjuvant chemotherapy for stage II and III colon cancer: Results from NSABP C-07. Journal of Clinical Oncology 25:2198–2204. https://doi.org/10.1200/JCO.2006.08.2974
20. Sadahiro S, Sakamoto K, Tsuchiya T, et al (2022) Prospective observational study of the efficacy of oral uracil and tegafur plus leucovorin for stage II colon cancer with risk factors for recurrence using propensity score matching (JFMC46-1201). BMC Cancer 22: https://doi.org/10.1186/s12885-022-09267-z
