# Supplementary material for: Evaluation of the prognostic impact of intraoperative cytology results according to tumor sidedness in stage II/III resectable colorectal cancer
Source: Int J Colorectal Dis. 2026 Jun 15;41(1):140. doi: 10.1007/s00384-026-05173-z (PMC13500429; doi:10.1007/s00384-026-05173-z)
Supplement: Supplementary file 2 [file 384_2026_5173_MOESM2_ESM.pdf]

STROBE Checklist（Cohort Study）

Title of Study:

Prognostic Impact of Intraoperative Lavage Cytology According to Tumor Sidedness in Stage II/III Resectable Colorectal Cancer

---

Title and Abstract

Item	Recommendation	Reported (Yes/No)	Page No.
1	Study design indicated in title/abstract	Yes	Title / Abstract
	Informative and balanced abstract	Yes	Abstract

Introduction

Item	Recommendation	Reported	Page No.
2	Scientific background and rationale	Yes	Introduction
3	Objectives and hypotheses clearly stated	Yes	Introduction

Methods

Item	Recommendation	Reported	Page No.
4	Study design presented early	Yes	Study design
5	Setting, locations, and dates	Yes	Study design
6	Participants eligibility criteria	Yes	Study design
	Methods of selection	Yes	Study design
7	Variables clearly defined	Yes	Methods / Data collection
8	Data sources and measurement	Yes	Data collection
9	Bias addressed	Partially	Discussion
10	Study size explained	No	—
11	Quantitative variables handling	Yes	Statistical analysis
12	Statistical methods described	Yes	Statistical analysis
	Confounding control	Partially	Statistical analysis
	Subgroup analysis	Yes	Results
	Missing data handling	No	—
	Loss to follow-up	Not clearly described	—

Results

Item	Recommendation	Reported	Page No.
13	Participant numbers at each stage	Yes	Results / Fig. 1
	Reasons for non-participation	Yes	Results
	Flow diagram	Yes	Fig. 1
14	Descriptive data	Yes	Table 1
	Missing data	No	—
	Follow-up time	Partially	Methods
15	Outcome data	Yes	Results
16	Main results with estimates and precision	Yes	Results
	Adjusted estimates	Partially	Table 3
17	Other analyses (subgroups)	Yes	Results

Discussion

Item	Recommendation	Reported	Page No.
18	Key results summarized	Yes	Discussion
19	Limitations discussed	Yes	Discussion
20	Interpretation consistent with results	Yes	Discussion
21	Generalizability discussed	Partially	Discussion

Other Information

Item	Recommendation	Reported	Page No.
22	Funding sources and role	Not described	—

Sample size

No formal sample size calculation was performed, as this study was a secondary analysis of a prospective registry.

Missing data

Missing data were minimal and handled using complete-case analysis.

Funding

This study received no external funding.
